# Silver Nanoparticles in Orthopedic Applications: New Insights on Their Effects on Osteogenic Cells

**DOI:** 10.3390/nano7060124

**Published:** 2017-05-27

**Authors:** Sara Castiglioni, Alessandra Cazzaniga, Laura Locatelli, Jeanette A. M. Maier

**Affiliations:** Dipartimento di Scienze Biomediche e Cliniche L. Sacco, Università di Milano, Milano I-20157, Italy; sara.castiglioni@unimi.it (S.C.); alessandra.cazzaniga@unimi.it (A.C.); laura.locatelli@unimi.it (L.L.)

**Keywords:** silver nanoparticles, osteoblasts, mesenchymal stem cells

## Abstract

Infections of orthopedic implants are associated with high morbidity. The emergence of antibiotic resistant strains and the tendency of microbes to form biofilms on orthopedic devices prompt the individuation of novel antimicrobial agents. Silver nanoparticles represent an interesting alternative, but their effects on bone cells need to be clarified. We focused on osteoblast-like cells and on bone marrow-mesenchymal stem cells and found that these cells are rather resistant to the cytotoxic effects of silver nanoparticles, with a half maximal inhibitory concentration around 25 µg/mL as detected by MTT assay. Within a month of treatment, osteoblast-like cells adapt to the presence of the nanoparticles by upregulating hsp70 as shown by western blot. Hsp70 overexpression correlates with the restoration of normal cell proliferation. No alterations in the extent and time requirements were detected in mesenchymal stem cell induced to differentiate in osteoblasts in the presence of silver nanoparticles. Because the concentrations of silver nanoparticles which show antimicrobial activity are lower than those exerting toxic effects on bone-forming cells in vitro, we suggest that silver nanoparticles might represent a challenging tool to fight infections in orthopedic implants.

## 1. Introduction

Thanks to the remarkable development of bioengineering, orthopedic implants are widely used to replace joints or to support damaged bones. Unfortunately, infections are relatively common and represent a major source of morbidity [1,2]. In particular, bacteria tend to generate biofilms on orthopedic devices and, once biofilms are formed, microbes resist immune attack and antibiotics [3]. This fact, in addition to the boost of antibiotic-resistant microorganisms, has triggered the development of novel antimicrobial agents. Because of their antiseptic effects, attention has been focused on metals, especially on metallic nanoparticles (NP), which are stable and provide a large surface area for contact with bacteria. Recently, silver nanoparticles (Ag NP) have emerged for their potent antiseptic properties [4] and are widely employed for domestic and medical devices. Ag NP show a marked bactericidal activity against Gram-positive and -negative bacteria and are effective also against antibiotic-resistant strains [4], because, after entering the bacteria, they inhibit enzymes and damage DNA and bacterial cell membranes [4]. On these bases, orthopedic implants coated with Ag NP have been developed and some of them are utilized in clinical trials [5].

However, concerns are raised about Ag NP biocompatibility. Ag NP cause abnormalities in zebrafish development in a dose-dependent manner [6] and are toxic for a variety of normal and neoplastic cells [7,8,9,10,11] mainly by generating oxidative and nitrosative stresses [9,10]. Since nanoparticulate silver coatings for orthopedic implants represent a promising approach to prevent infections, the effects of Ag NP have been studied on bone cells. Ag NP are toxic for osteoblasts [12,13,14], specialized bone forming cells, and osteoclasts [12], which resorb the bone. In the case of osteoblasts, toxicity has been linked to the activation of inducible nitric oxide synthase (iNOS) with consequent accumulation of nitric oxide and reactive nitrogen species, which have deleterious effects on human osteoblast metabolism [14]. In addition, controversial results are available about mesenchymal stem cells (MSC), the multipotent precursors of osteoblasts. Ag NP impair the viability of human MSC (hMSC) in a dose- and time-dependent fashion, likely through the induction of DNA damage [15], while they do not influence their differentiation [16]. Another study, however, shows that Ag NP, which enter the cells through endocytosis and then accumulate in the cytosol, attenuate both osteogenic and adipogenic differentiation of hMSC, even at non-toxic concentrations [17]. On the contrary, Ag NP stimulate the osteogenic differentiation of murine MSC by activating TGF beta signaling [18].

It is very difficult to summarize data about the effects of Ag NP on cultured cells because the experiments are performed with Ag NP of different size, used at different concentrations and for different times. However, all the results available agree on the fact that Ag NP are toxic to many cell types in a dose dependent manner.

In this study, we have investigated the behavior of Saos-2 osteoblast-like cells [19] exposed to various concentrations of Ag NP for different times. These cells have been widely used to study bone formation, osteoporosis and also for biomedical applications [20,21,22] since they show a mature osteoblastic phenotype. Because Ag NP and hMSC are used in bone tissue engineering, we also studied how hMSC [23] respond to Ag NP in terms of viability and osteogenic differentiation.

## 2. Results

### 2.1. Ag NP Reduce Viability and Increase ROS Production in Saos-2

**Figure 1:**
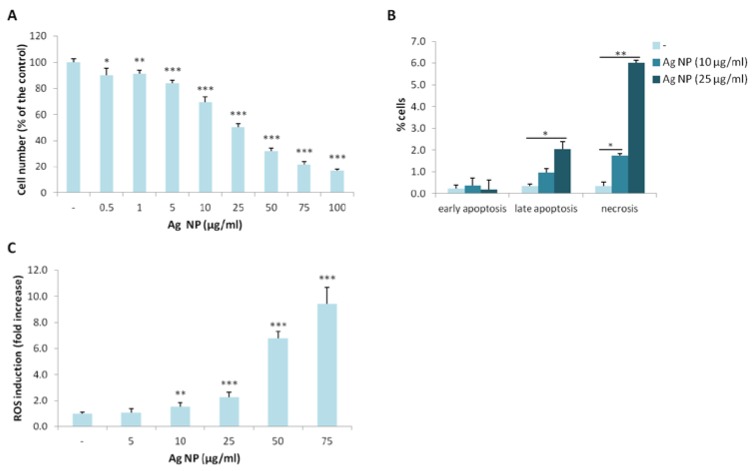
Ag NP are cytotoxic for Saos-2. (**A**) Saos-2 were cultured for 5 days with Ag NP (0.5–100 µg/mL). Viability was assessed by MTT assay. Data are expressed as the percentage of control (-). *p* value was calculated vs. untreated cells: * *p* < 0.05, ** *p* < 0.01, *** *p* < 0.001; (**B**) Saos-2 were treated for 24 h with Ag NP (10 and 25 μg/mL). After staining with Annexin V/PI flow cytometry was performed. % of the cells in early, late apoptosis or necrosis were shown in the graphic. Data are the means ± standard deviation (SD) of three separate experiments. *p* value was calculated vs. untreated cells (-): * *p* < 0.05, ** *p* < 0.01; (**C**) After 24 h of culture in the presence of Ag NP, ROS production was measured. Results are shown as the fold increase of ROS in Ag NP treated cells vs. untreated control (-). Data are the means ± SD of three separate experiments in triplicate. *p* value was calculated vs. untreated cells: ** *p* < 0.01, *** *p* < 0.001.

We investigated the toxicity of Ag NP on Saos-2 by culturing them in the presence of different concentrations of Ag NP for five days. By MTT assay, we found that Ag NP reduce the viability of Saos-2 in a dose-dependent manner with a half maximal inhibitory concentration (IC_50_) around 25 µg/mL (Figure 1A). We then performed the Annexin V/PI staining and found that 10 and 25 µg/mL Ag NP increases cell death after 24 h. In particular, a significant increase of late apoptotic phase was detected with 25 µg/mL. After exposure to 10 and 25 µg/mL Ag NP, a marked increase of necrotic cells was detected (Figure 1B). Since several studies link Ag NP toxicity with the accumulation of reactive oxygen species (ROS), we quantified ROS levels after exposing Saos-2 to Ag NP for 24 h and detected a significant dose-dependent increase of ROS starting from 10 µg/mL (Figure 1C). It is noteworthy, however, that cytotoxicity occurs at lower concentrations, possibly induced by other mechanisms.

Similar results were obtained when we utilized PolyVinylPyrrolidone (PVP)-coated Ag NP (not shown), as previously shown in another cell type [8], thus ruling out a role of Ag ions in causing toxicity.

### 2.2. Saos-2 Adapt to Long Term Exposure to Ag NP

**Figure 2:**
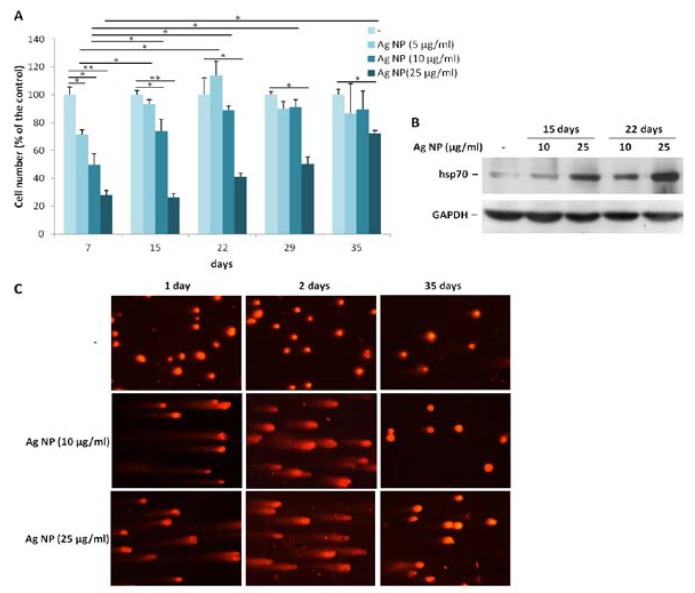
Saos-2 adapt to long term exposure to Ag NP. (**A**) Saos-2 cells were treated with Ag NP (5, 10, and 25 µg/mL). On the day in which the untreated control (-) became confluent, all the cells were counted and re-seeded at the same density. Data are expressed as the percentage relative to seven days-untreated cells. Data represent the means ± SD of three separate experiments in triplicate (* *p* < 0.05, ** *p* < 0.01); (**B**) Saos-2 were exposed to Ag NP (10 and 25 μg/mL) for 15 and 22 days. Western blot was performed using anti-hsp70 antibodies. GAPDH was used as a control of sample loading. (-: untreated cells); (**C**) Saos-2 were treated with Ag NP (10 and 25 μg/mL) for 1, 2, and 35 days. Comet assay was performed after staining with ethidium bromide and analyzed with a fluorescence microscope. (-: untreated cells).

To evaluate the effects of long term exposure to Ag NP, we treated Saos-2 with three different concentrations of Ag NP (5, 10, and 25 µg/mL) for more than a month. All the cells were trypsinized and counted as soon as the control untreated cells reached confluence. Then, the cells were seeded again at the same density. As shown in Figure 2A, after seven days of treatment, cell growth was severely impaired at all the concentrations tested. The proliferation of Saos-2 exposed to 5 or 10 µg/mL was restored to levels comparable with the untreated controls after 15 and 22 days, respectively, from the beginning of the experiment. Only after 35 days the toxic effect of 25 µg/mL of Ag NP was significantly reduced. We reasoned that, upon long term exposure to Ag NP, the cells become resistant to the toxic effects of Ag NP, possibly by activating a stress response. To test this issue, Saos-2 were treated for 15 and 22 days with Ag NP (10 and 25 µg/mL) and a western blot was performed on lysates using anti-hsp70 antibodies. Figure 2B shows a time- and dose-dependent upregulation of hsp70.

We also investigated whether exposure to Ag NP caused DNA strand breaks in Saos-2 by comet assay. As demonstrated in Figure 2C, 1 and 2 days of Ag NP treatment induced genotoxic stress as shown by the formation of comets. Comet assay was also performed on Saos-2 treated 35 days with Ag NP. Figure 2C (right panel) shows that genotoxicity was completely reversible upon long term exposure to NP at the concentration of 10 µg/mL, whereas in Saos-2 exposed to 25 µg/mL of Ag NP genotoxicity was only partially reversible.

### 2.3. Ag NP Do Not Impair the Differentiation of hMSC

**Figure 3:**
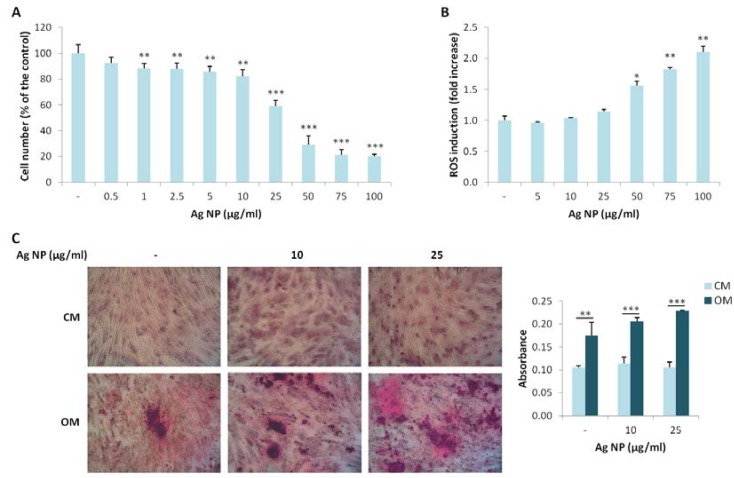
Ag NP do not impair hMSC differentiation into osteoblasts. (**A**) hMSC were treated for 5 days with Ag NP (0.5–100 µg/mL) and MTT assay was performed to assess viability. Data are expressed as the percentage of control (-). *p* value was calculated vs. untreated cells: ** *p* < 0.01, *** *p* < 0.001; (**B**) hMSC were exposed to Ag NP (5–100 µg/mL) for 24 h and ROS generation was measured. Data are shown as the fold increase in ROS levels of NP treated cells compared to control (-). *p* value was calculated vs. untreated cells: * *p* < 0.05, ** *p* < 0.01; (**C**) hMSC were cultured in CM or OM for 15 days in the presence of Ag NP (10 and 25 μg/mL) and stained with Alizarin Red S. Photographs were taken at 10× magnification. After acid extraction, the absorbance was measured at 562 nm. Data are represented as the means ± SD of three separate experiments in triplicate: ** *p* < 0.01, *** *p* < 0.001. (-: untreated cells).

We extended our studies to hMSC, which possess the potential to differentiate into different cell types among which osteoblasts. We investigated the effects of various concentration of Ag NP. As demonstrated for Saos-2, Ag NP-induced cytotoxicity is dose dependent (Figure 3A). Similarly to Saos-2, the IC_50_ is around 25 µg/mL. We then analyzed ROS induction after Ag NP treatment and observed a statistically significant increase of ROS production only at high NP concentrations (50, 75, and 100 µg/mL) (Figure 3B).

Finally, we studied the effects of Ag NP on the osteogenic differentiation of hMSC. The cells were cultured in their culture medium (CM) or in a medium containing an osteogenic cocktail (OM) in the presence of 10 and 25 µg/mL of Ag NP. Alizarin Red S staining shows a significant increase of calcium deposits in the cells cultured in OM independently from the treatment with Ag NP (Figure 3C), thus indicating that Ag NP do not interfere with the response of the cells to the osteogenic cocktail.

## 3. Discussion

Because (i) postoperative infections of orthopedic implants lead to the failure of fixation and are associated with high morbidity, and (ii) antibiotic resistant bacteria are rapidly emerging, Ag NP might represent a good candidate for coating these devices, thereby circumventing bacterial colonization [24]. However, the use of Ag NP is debated because of their toxicity in vivo and in many cell types in vitro [6,7,8,9,10,11], even though in several cases the underlying mechanisms have not been elucidated. Initially, we focused on human osteoblast-like cells and found a dose dependent toxic effect of Ag NP, with an IC_50_ around 25 µg/mL. Previous studies on human fetal osteoblasts have demonstrated an impairment of cell viability with 30–60 µg/mL Ag NP after 24 and 48 h because of the increase of iNOS [14]. Differently from [14], we did not detect any significant modulation of nitric oxide synthesis after exposure to Ag NP from 0.5 to 100 µg/mL for 24 and 48 h (data not shown). It should be noted that discrepancies could be attributed, at least in part, to the different treatment protocols used, to NP composition, and to their different size. Ag NP used in [14] are 18–20 nm in size, while ours are around 30 nm, and it is well accepted that an inverse correlation exists between cytotoxicity and NP size [25].

In Saos-2, after the addition of Ag NP, we detected an increase of ROS and oxidative damage to the DNA. These results are in agreement with a large part of the literature indicating ROS as important mediators of Ag NP cyto- and geno-toxicity [7]. Indeed, Ag NP impair the mitochondrial respiratory chain with consequent accumulation of ROS and reduced synthesis of ATP [7]. However, it is noteworthy that 5 µg/mL Ag NP are cytotoxic without inducing ROS accumulation. Therefore, the accumulation of ROS might contribute to cytotoxicity only at high concentrations of Ag NP and other undisclosed mechanisms are involved in mediating the effects of Ag NP in Saos-2.

It is relevant to underscore that, over time, Saos-2 adapt to the presence of Ag NP through the activation of a stress response. We found that Saos-2 cultured in the presence of sublethal concentrations of Ag NP upregulate hsp70 and this parallels the decrease of oxidative damage to DNA and the gradual recovery of cell proliferation. Indeed, in addition to its primary action as a chaperone, hsp70 interacts with crucial regulators of many signal transduction pathways controlling cell proliferation, differentiation, and death [26]. High levels of hsp70 protect against apoptosis by inhibiting caspase-dependent and independent pathways. Moreover, hsp70 is important in the control of cell cycling and growth and, accordingly, some oncogenes upregulate hsp70 [26]. We conclude that Saos-2 adapt and resist to Ag NP because of the upregulation of hsp70.

hMSC show a response to Ag NP that is very similar to that of Saos-2. The IC_50_ is around 25 µg/mL and the overproduction of ROS is observed with 50 µg/mL and over. Again, we argue for alternative pathways responsible for cytotoxicity. Interestingly, Ag NP do not influence the differentiation of hMSC towards osteoblasts. Our results are in accordance with a previous report [16]. The different size of nanoparticles used as well as the different experimental protocol might explain the different results obtained by Sengstock et al. [17], who showed an attenuation of adipogenic and osteogenic differentiation in hMSC treated with 80 nm size Ag NP.

Both hMSC and Saos-2 seem to be rather resistant to the toxic effect of Ag NP if compared with cell types we previously used. Bladder carcinoma T24 and microvascular endothelial cells showed an IC_50_ around 7.5 and 2.5 µg/mL Ag NP, respectively [8,9]. In the light of a use for orthopedic implants, it should be recalled that the concentrations of Ag NP required to obtain bactericidal effects are lower than those which are toxic for bone cells [27,28]. Additionally, it is likely that the incorporation of Ag NP in biomaterials might further decrease cytotoxicity on eukaryotic cells.

Another challenging issue that has recently emerged is the demonstration of a gender-dependent sensitivity to NP of different sizes. Lee and colleagues [29] reported a longer persistence of Ag NP in the blood of female Sprague-Dawley rats after oral administration of high concentrations of 25 nm Ag NP. On the contrary, a longer persistence of 10 nm Ag NP was reported in male rats. This result might be hormone-dependent. Cultured cells represent a simple and useful preclinical model to decipher the mechanisms of Ag NP toxicity, but cannot provide any hint about gender differences.

In conclusion, we suggest that Ag NP might be used as antimicrobial materials, without compromising the behavior of bone-forming cells.

## 4. Materials and Methods

### 4.1. Reagents

Spherical Ag NP with an average size of 35 nm (#0476JY, NanoAmor, Houston, TX, USA) were previously used [8]. They have surface area of 30–50 m^2^/g. Stock solution of these NP was suspended in Dulbecco’s Modified Eagle’s Medium (DMEM) at a concentration of 1080 mg/mL. We have assessed that NP stock solution can be maintained at 4 °C for one month without altering NP properties (not shown). This solution was sonicated many times just before its dilution in culture medium.

### 4.2. Cell Culture

Saos-2 (American Type Culture Collection) were cultured in DMEM with 10% fetal bovine serum and 2 mM glutamine at 37 °C and 5% CO_2_. hMSC were isolated from adult human bone marrow [23] according to IRCCS Policlinico institutional guidelines and characterized by flow cytometry. These cells were cultured at 37 °C and 5% CO_2_ in DMEM with 1000 mg/L glucose, 10% FBS, and 2 mM glutamine (culture medium, CM). All reagents were purchased from Sigma-Aldrich (St. Louis, MO, USA).

### 4.3. Cell Viability Assay

To test the effects of Ag NP, we used the MTT assay. Saos-2 and hMSC were seeded at 6500/cm^2^ in 96 well/plates and 24 h later exposed to Ag NP. After five days of treatment, 3-(4,5-Dimethylthiazol-2-yl)-2,5-Diphenyltetrazolium Bromide was added (MTT, 0.5 mg/mL) (Sigma-Aldrich, St. Louis, MO, USA) as described [9]. Formazan crystals were dissolved in DMSO and absorbance was measured at 550 nm.

In long-term experiments, Saos-2 (6500/cm^2^) were seeded in 24 well/plates 24 h prior to treatment with Ag NP. When the untreated cells were confluent, the cells were trypsinized, counted using an automated counter, and seeded (6500/cm^2^) in a new 24-well plate in the presence of the correspondent concentrations of Ag NP. Data represent the mean ± standard deviation of three separate experiments in triplicate.

### 4.4. Flow Cytometric Analysis of Cell Death

The effect of Ag NP on cell death was investigated by Annexin V-FITC and propidium iodide (PI) staining as described [8]. Briefly, Saos-2 cells were harvested, extensively washed, centrifuged at 1200 rpm for 5 min and stained for 30 min with 5 mL of Annexin V-FITC and 5 mL PI according to the manufacturer’s protocol (Immunostep, Salamanca, Spain). The samples were then evaluated by cytofluorimetry using the FACScalibur (BD Biosciences, Singapore) at 10,000 events for each sample [8].

### 4.5. Reactive Oxygen Species Production

The levels of intracellular ROS were measured using 2′-7′-dichlorofluorescein diacetate (DCFH) as previously described [8,9]. Cells seeded in black bottomed 96-well plates were exposed to different concentrations of Ag NP. 24 h later, 20 µM DCFH solution were added to the culture media. The rate of ROS production was monitored following the emission at 529 nm of the DCFH dye with Promega Glomax Multi Detection System [8,9]. Three independent experiments in triplicate were performed and the results are shown as the fold increase in ROS levels of Ag NP-treated cells vs. the control ± standard deviation.

### 4.6. Comet Assay (Single Cell Electrophoresis)

Saos-2 were seeded in 24-well plates 24 h prior to exposure to 10 and 25 ug/mL of Ag NP. Untreated cells were the controls. At the end of the experiment, cells were trypsinized, mixed with low melting-point agarose, and spread on agarose pretreated slides as described [9]. Slides were dried and immersed in lysis solution [9] at 4 °C for 60 min. Electrophoresis was performed for 30 min at 300 mA in ice cold running buffer [9]. Then, slides were rinsed in Tris-HCl pH 7.5, fixed in ice-cold methanol for 3 min, dried at room temperature, stained with ethidium bromide, and analyzed using a fluorescence microscope.

### 4.7. Western Blot Analysis

Lysates (100 µg/lane) were run on a 10% SDS-polyacrylamide gel, transferred to nitrocellulose sheets for 16 h at 150 mA, and probed with antibodies against hsp70 or GAPDH (Santa Cruz Biotechnology, Dallas, TX, USA). Secondary antibodies were labelled with horseradish peroxidase (GE Healthcare, Little Chalfont, UK). Immunoreactive proteins were visualized by the SuperSignal chemiluminescence kit (Pierce, Thermo Fischer scientific, Monza, Italy). The experiment was repeated three times and one representative blot is shown.

### 4.8. In Vitro Osteogenic Differentiation of hMSC

Confluent hMSC were cultured in their culture medium (CM) or in a medium containing an osteogenic cocktail (OM) in the presence of 10 and 25 µg/mL of Ag NP. The OM contains 2 × 10^−8^ M 1α,25-Dihydroxyvitamin D_3_, 10 mM β-glycerolphosphate and 0.05 mM ascorbic acid (Sigma-Aldrich, St. Louis, MO, USA). To detect calcium in the extracellular matrix, hMSC were rinsed with phosphate buffered saline, fixed (70% ethanol, 1 h), and stained with 2% Alizarin Red S staining (pH 4.2, Sigma-Aldrich, St. Louis, MO, USA) for 10 min [19]. Quantification of calcium deposition was performed after solubilization in 10% cetylpyridinium chloride (Sigma-Aldrich, St. Louis, MO, USA) and resuspended in 10 mM sodium phosphate (pH 7.0). The absorbance was measured at 562 nm.

### 4.9. Statistical Analysis

Student’s *t*-test was used for calculating Statistical significance. * *p* < 0.05, ** *p* < 0.01, *** *p* < 0.001.
